# Multimodal Second‐Harmonic‐Generation, Two‐Photon Excitation Fluorescence, and Brillouin Microscopy for Visualising Dermal Mechanical Properties in Ex Vivo Human Skin

**DOI:** 10.1111/exd.70081

**Published:** 2025-03-12

**Authors:** Eiji Hase, Naoya Okubo, Yuki Ogura, Takeo Minamikawa, Takeshi Yasui

**Affiliations:** ^1^ Institute of Post‐LED Photonics Tokushima University Tokushima Tokushima Japan; ^2^ Graduate School of Sciences and Technology for Innovation Tokushima University Tokushima Tokushima Japan; ^3^ Shiseido Global Innovation Center Yokohama Kanagawa Japan; ^4^ Graduate School of Engineering Science Osaka University Toyonaka Osaka Japan

**Keywords:** Brillouin microscopy, mechanical property, optical microscopy, second‐harmonic‐generation microscopy, two‐photon excitation fluorescence microscopy

Multiphoton microscopy, particularly second‐harmonic‐generation (SHG) and two‐photon excitation fluorescence (TPEF) microscopy, has been a powerful tool for observing the structural and compositional remodelling of the dermal extracellular matrix (ECM) as it selectively visualises collagen and elastic fibres [1]. Therefore, combining data from multiphoton microscopy with mechanical tests offers valuable insights into how ECM remodelling resulting from disease or ageing affects the mechanical properties of the dermis. Although various mechanical tests such as rheology [2], atomic force microscopy‐based microindentation [3], and ultrasound [4] are commonly used to assess mechanical properties, their spatial resolution or field of view often do not align with that of multiphoton microscopy, limiting the integration of findings related to ECM remodelling and mechanics.

In this study, we used Brillouin microscopy [5], which provides information about the viscoelastic properties of biological tissues with sub‐micrometre, three‐dimensional spatial resolution in a label‐free, non‐contact manner, and combined it with multiphoton microscopy as an alternative to traditional mechanical tests.

We integrated multiphoton microscopy and Brillouin microscopy into a single microscope, allowing switching between them (Figure S1). A femtosecond optical parametric oscillator (central wavelength = 800 nm, pulse duration ≈ 110 fs, repetition rate = 80 MHz) and the laser scanning system were used to capture SHG and TPEF images, visualising the distribution of collagen and elastic fibres, respectively. For Brillouin microscopy, a single‐mode diode‐pumped solid‐state laser (wavelength = 532 nm) was used, and the Brillouin spectra were measured by a tandem VIPA spectrometer and an EMCCD camera (exposure time = 100 ms). A normal abdominal skin sample from a healthy Caucasian female, provided by Obio LLC (CA, USA), was embedded in optimal cutting temperature (OCT) compound, flash‐frozen, and stored at −80°C. An 8 μm‐thick section was then analysed using multimodal microscopy. To mimic physiological conditions, the sample was kept moist by applying phosphate‐buffered saline (PBS) solution.

Figure 1a shows a bright‐field image of the sample, with the red square indicating the dermal area measured by multimodal microscopy. The multiphoton imaging results, shown in Figure 1b,c, visualised that both collagen and elastin exist on fibres, with collagen being more densely distributed compared to the sparser distribution of elastin. The optical frequency shift of Brillouin scattering, namely Brillouin shift, is proportional to the longitudinal modulus, which is defined as the ratio of uniaxial stress to strain under a longitudinally confined condition [5]. Therefore, the Brillouin image shown in Figure 1d reflects the distribution of the local mechanical property linked to elasticity. From the comparison with the multiphoton images, we observed that areas with a high Brillouin shift contain a higher concentration of collagen relative to elastin. To evaluate these relationships more quantitatively, we performed a pixel‐by‐pixel correlation analysis of the Brillouin image and SHG or TPEF images, as shown in Figure 1e,f. From the comparison between them, the correlation between the Brillouin shift and SHG intensity showed a higher coefficient of determination (*R* ^2^ = 0.29), which may imply that collagen, rather than elastin, plays a greater role in influencing the elasticity observed through the Brillouin shift. This aligns with previous studies [6] where ECM components were isolated for mechanical testing, demonstrating that collagen exhibits a higher elastic modulus than elastin. Moreover, the fibrous structures in the Brillouin image likely correspond to collagen fibres, as indicated by the comparison with SHG images. These findings demonstrate the potential of our multimodal microscopy to locally measure the mechanical properties of fibrous ECM at micrometre‐scale resolution. Future studies will investigate differences in collagen fibre organisation between dermal layers using SHG imaging, focusing on how thinner, loosely arranged fibres in the papillary dermis and thicker, densely packed fibres in the reticular dermis may contribute to depth‐dependent variations in elasticity observed in the Brillouin shift. While these potential relationships suggest a link between collagen morphology and mechanical properties, understanding the relationship between Brillouin shift and SHG intensity still requires further investigation. SHG intensity depends not only on the square of collagen density but also on additional factors such as the structural maturity of collagen fibres [1]. Combining polarisation‐resolved SHG microscopy [7] could help clarify how the microstructural properties of collagen relate to the observed mechanical properties. It is also important to note that the Brillouin shift is influenced by water content [5], and the presence of proteoglycans in the skin may further complicate this relationship. Future research should investigate how these non‐collagenous, non‐elastin components, particularly proteoglycans and their water‐binding capacity, influence Brillouin measurements and how this might affect the understanding of dermal mechanical properties.

**FIGURE 1:**
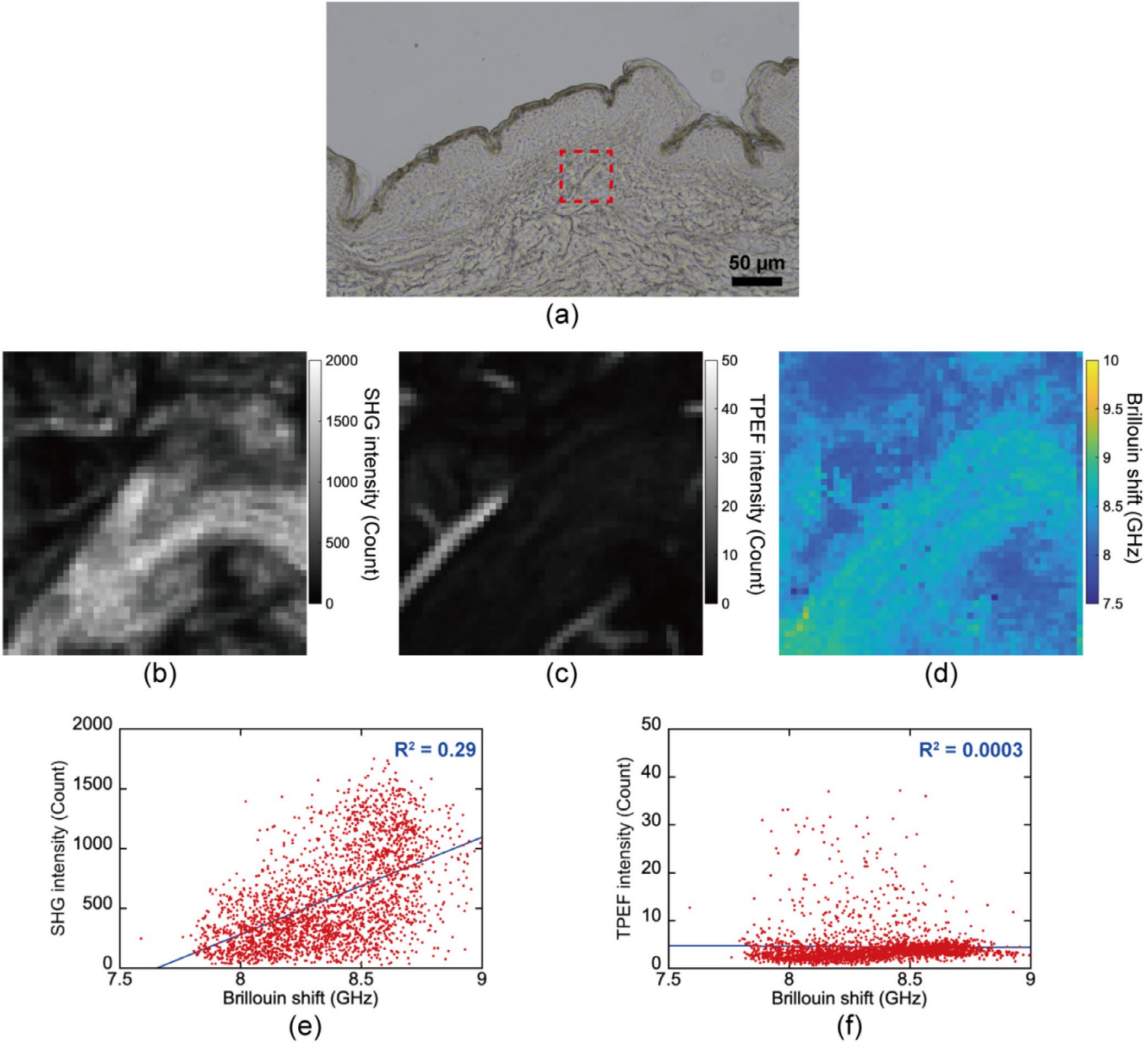
Multimodal imaging results of ex vivo human dermis. (a) Bright‐field image of the skin sample. (b) SHG image, (c) TPEF image, and (d) Brillouin image acquired from the region indicated by the red dashed square in (a). The image size is 50 × 50 pixels, corresponding to 50 × 50 μm. (e) Pixel‐by‐pixel correlation between the Brillouin shift and SHG intensity, and (f) between the Brillouin shift and TPEF intensity. Red dots represent the measured values, while the blue lines indicate linear fits.

Despite the complexities, the ability to assess both structural and mechanical changes in collagen and elastin at micrometre‐scale resolution makes the proposed multimodal microscopy a promising tool for analysing ECM disorders characterised by mechanical property abnormalities. This includes conditions such as Ehlers‐Danlos syndrome, Marfan syndrome, scleroderma, and age‐related changes, all of which involve significant collagen and elastic fibre remodelling. Future refinements in the correlation between modalities will provide a deeper understanding of how ECM components contribute to dermal mechanical properties, potentially enabling in vivo applications. However, extending these capabilities to in vivo studies will require further advancement in imaging technology, including faster acquisition and motion correction techniques.

## Author Contributions

E.H. was involved in the conceptualisation and design of the study and wrote the manuscript. E.H. and N.O. constructed the system. E.H., N.O., and Y.O. were involved in data acquisition and analysis. N.O. and Y.O. handled sample preparation. T.M. and T.Y. contributed to data interpretation. Y.O. and T.Y. revised the manuscript. All authors approved the final version to be published.

## Conflicts of Interest

The authors declare no conflicts of interest. However, Yuki Ogura is employed by Shiseido Co. Ltd, Japan, which may have a commercial interest in the research findings.

## Supporting information


Appendix S1.

## Data Availability

The data supporting this study's findings are available from the corresponding author upon reasonable request. The skin sample used in this study was obtained from Obio LLC (CA, USA), which confirmed compliance with all relevant laws, ethical codes, and regulations. The study protocol was also approved by the ethical review board at Tokushima University (No. 14003).
