# Supplementary material for: Multimodal Second‐Harmonic‐Generation, Two‐Photon Excitation Fluorescence, and Brillouin Microscopy for Visualising Dermal Mechanical Properties in Ex Vivo Human Skin
Source: Exp Dermatol. 2025 Mar 12;34(3):e70081. doi: 10.1111/exd.70081 (PMC11903916; doi:10.1111/exd.70081)
Supplement: Supplementary file 1 — Appendix S1. [file EXD-34-e70081-s001.docx]

**SUPPORTING INFORMATION:**

**Experimental setup of multimodal optical microscopy:**

Supplemental Figure 1 shows the experimental setup for multimodal optical microscopy. Based on our previous study [S1], we constructed a laser-scanning SHG and TPEF microscope using a femtosecond optical parametric oscillator (femtosecond OPO; InSight X3+, MKS Instruments, Inc., Milpitas, CA, US; tuning range = 680–1300 nm; pulse duration ≈ 110 fs; repetition rate = 80 MHz). The laser polarization was set to the circular polarization to cancel out the polarization dependency of the SHG intensity. The focus spot of the laser beam is scanned two-dimensionally onto a sample using a pair of galvanometer mirrors (GMs), a pair of relay lenses, and an objective lens (OL; CFI S Plan Fluor ELWD 40XC, Nikon, Corp., Tokyo, Japan; NA = 0.6, magnification = 40). The backscattered SHG and TPEF light was separated by dichroic mirrors (DM1; FF705-Di01, Semrock, Inc., Rochester, NY, US, DM2; FF409-Di03, Semrock, Inc., Rochester, NY, US) and optical filters (BPF1; FBH400-10, Thorlabs, inc., Newton, NJ, US, BPF2; FBH450-40, Thorlabs, inc., Newton, NJ, US). Finally, SHG and TPEF light were individually detected by the photon-counting photomultipliers (PC-PMT1; H10682-210-01, Hamamatsu Photonics, KK., Shizuoka, Japan, PC-PMT2; H8259, Hamamatsu Photonics, KK., Shizuoka, Japan) connected to a pulse counter. The pixel dwell time was 125 µs/pixel.

For Brillouin microscopy, a single-mode diode-pumped solid-state laser (DPSS laser; Torus 532, Laser Quantum Ltd., Stockport, UK; λ = 532 nm, Δν < 1 MHz, P = 200 mW) was used as the light source. The light source was placed on a customized fan-cooled heat sink (Pneum Co., Ltd., Saitama, Japan) to minimize temperature drift of oscillation wavelength. Brillouin scattering light backscattered in the sample was delivered via a single-mode fibre (SMF) while giving the confocality, and then was fed into a tandem VIPA spectrometer [S2, S3]. Following previous studies [S2, S3], we constructed the tandem VIPA spectrometer consisting of two identical VIPAs (OP-6721-3371-2, Light Machinery, Inc., Nepean, Ontario, Canada; FSR = 30 GHz, Finesse = 65) arranged orthogonally, 4-f optics of relay lenses, and two orthogonal slits and spatial filter (also known as Lyot filter) to block the strong background of Rayleigh scattering light. A sharp optical bandpass filter (BPF3; LL01-532-12.5, Semrock, Inc., Rochester, NY, US) was further placed to reject the unnecessary Raman scattering light. The spatially dispersed Brillouin scattering light was then measured by an EMCCD camera (iXon Life 897, Andor Technology Ltd., Belfast, UK; pixel size = 512 × 512 pixels). The exposure time and EM gain of the EMCCD camera were set to 100 ms and 200, respectively. Two-dimensional imaging was performed by scanning the XY translation stage in the microscope. The Brillouin spectrum at each pixel was analysed without the signal accumulation.

Both microscopy setups were built within the same microscope body (Ti2-E, Nikon, Corp., Tokyo, Japan), and the same objective lens was used for both modalities. Each laser beam was guided to the different side ports on the microscope, as shown in Fig. S1, and the optical path was switched using the flipper mirror (FM) inside the microscope. Multiphoton and Brillouin measurements were individually controlled by a custom LabVIEW program, which integrates a software library for stage control (Science Edge Co., Ltd., Hamamatsu, Japan) and camera control (Andor Technology Ltd., Belfast, UK).

While this study focused on *ex vivo* imaging, the potential for *in vivo* applications remains an important future direction. However, extending these capabilities to *in vivo* studies will require further advancements in imaging technology, including faster acquisition and motion correction techniques [S4, 5].

**Spectral analysis in Brillouin microscopy:**

In Brillouin microscopy, we calculated the optical frequency shift *Ω* in the Brillouin spectrum (Brillouin shift) at each sample location.

$$\begin{aligned} \Omega=2\sin\left( \frac{\theta}{2} \right)\frac{n\omega_{i}}{c}v.\#\left( 1 \right) \end{aligned}$$

Here, *θ* represents the angle between the incident and scattered light (When employing reflection geometry, *θ* is 180˚), *n* is the refractive index, *ω_i_* is the optical frequency of the incident light, *c* is the speed of light in vacuum, and *v* is the speed of sound [S6]. Additionally, *v* can be expressed as follows:

$$\begin{aligned} v=\sqrt{\frac{M^{'}}{\rho}}.\#\left( 2 \right) \end{aligned}$$

*M'* denotes the longitudinal modulus, reflecting the material's local elasticity under a longitudinally confined condition, while *ρ* stands for density [S6]. Therefore, using Brillouin microscopy in combination with density and refractive index measurement [S7], it is possible to obtain the local mechanical properties without external mechanical load. Even without knowledge of the density or refractive index, it is possible to extract useful mechanical information, as the ratio between the refractive index and density tends to remain constant or cancel out in most cases [S6, S8]. However, in biological samples, spatial unevenness of this ratio exists, leading to inaccuracies in the Brillouin shift [S6]. In such a case, the validity of the Lorentz-Lorenz equation, which describes the relationship between refractive index and density, becomes particularly important [S8]. If this relationship is not valid, it could lead to discrepancies in the mechanical properties obtained from Brillouin microscopy. While this study treated the Brillouin shift as a value reflecting the elastic modulus, future investigations should apply Brillouin microscopy in combination with refractive index and density measurements as well as test the validity of the Lorentz-Lorenz equation in collagen and elastin. These approaches could help improve the interpretation of mechanical properties obtained by Brillouin microscopy in complex tissues like the skin.

In order to determine the Brillouin shift, we applied the nonlinear least-squares curve fitting analysis using a custom MATLAB script to both the Stokes and Anti-stokes Brillouin components with the sum of two Lorentzian functions. Before the measurement, we calculated the actual FSR (free spectral range) of VIPA and the pixel-to-frequency conversion ratio (PR) on the EMCCD camera using the measured results with ethanol and water, of which the Brillouin shifts are known [S3, S9]. In our experiment, FSR and PR were determined to be 28.25 GHz and 0.1075 GHz/pixel, respectively. Then, we calculated the Brillouin shift from the distance between the Stokes and Anti-stokes components [S3]. Supplemental Figure 2 shows the typical spectrum in the dermis obtained by our system. The Brillouin spectrum in the dermis has a single pair of Stokes and Anti-Stokes peaks, which is consistent with observations from a previous study [S5]. By repeating this calculation for each pixel, a Brillouin image was obtained.

**Image acquisition at the same location with multimodal microscopy:**

To perform pixel-by-pixel correlation analysis using Brillouin and multiphoton images, we ensured that measurements were taken at the same location on the sample. Under our experimental conditions, the image size in multiphoton microscopy was set to 400 × 400 pixels, corresponding to an actual area of 215 µm × 215 µm, while the Brillouin image was set to 50 × 50 pixels, covering 50 µm × 50 µm. To identify the matching region between the two images, we performed a two-dimensional (2D) cross-correlation-based image registration using a custom MATLAB script. For comparison between the images obtained by the different optical contrasts, we used a fluorescence image acquired through the optical path of the Brillouin microscope [Fig. S3(a)] and a TPEF image from the multiphoton microscope [Fig. S3(b)]. Both images were acquired using same fluorescent microspheres placed on calibration slides (StarLight Calibration Slides Envy Green, Polysciences, Inc., Warrington, PA, USA). The central wavelength of the femtosecond laser was changed to 1050 nm to optimize excitation efficiency, and we confirmed that variations in the excitation wavelength did not affect the lateral positioning on the focal plane. The TPEF image was detected using the above optical setup with a different optical bandpass filter (FF01-565/133-25, Semrock, Inc., Rochester, NY, US), and the fluorescence signal from the Brillouin microscope optical path (with 532 nm excitation) was detected using a PMT (H10723-110, Hamamatsu Photonics, KK., Shizuoka, Japan) and another optical bandpass filter (FBH560-10, Thorlabs, inc., Newton, NJ, US) placed before the first cylindrical lens (CL) in the tandem VIPA spectrometer (Both PMT and optical filter were not shown in Fig. S1.). The BPF3 was temporarily removed during this measurement. To apply the 2D cross-correlation-based image registration, the fluorescence image obtained through the optical path of the Brillouin microscope was matched against different regions of the TPEF image, which covered a larger field of view. Due to the difference in pixel-to-real-size ratios between the images, sub-images were extracted from the TPEF image [Fig. S3(c)] by varying the cropping area and scaling factor. The scaling factor adjustments were made using bilinear interpolation. The cross-correlation coefficient was then calculated between the fluorescence image and each sub-image, with the region of highest correlation identified [Fig. S3(d)]. The corresponding starting coordinates (x, y) for cropping and scaling factor were recorded and used for the multimodal imaging and the pixel-by-pixel analysis. The limitation of this method is that we did not take into account the refractive index dispersion of the sample to be measured by multimodal microscopy. This effect is particularly significant in the optical axis (z-direction), resulting in the acquisition of images at different focal planes, depending on the modality (i.e., due to the difference in the laser wavelength). In this experiment, we used a thin sample and adjusted the focus for each modality to the depth where the Brillouin and SHG signals were maximized. In the future, it will be necessary to consider methods such as adding markers to the sample itself [S10] to improve the correlation within the multimodal system.

**Sample preparation:**

A normal human abdominal skin sample was obtained from a healthy Caucasian female, provided by Obio, LLC (CA, USA), and embedded in optimal cutting temperature (OCT) compound before being flash-frozen in liquid nitrogen. The samples were stored at -80°C until sectioning. Thin sections (8 µm) were cut using a cryostat and immediately rinsed with phosphate-buffered saline (PBS, pH 7.4) solution to remove residual OCT. The sections were kept in PBS solution during imaging to maintain hydration and osmotic balance.

Since this study focused on fibrous structures such as collagen and elastin, which are mechanically robust compared to cellular components, the impact of freezing and sectioning on the measured properties is considered minimal. Bright-filed imaging was performed before multimodal imaging to verify the structural integrity of the sections, confirming that the fibrous structures remained intact.

Given that this study serves as a proof-of-concept for multimodal Brillouin, SHG, and TPEF imaging, sectioned samples were used to provide a controlled and well-characterized environment. In Brillouin microscopy, Rayleigh scattering in bulk skin tissue can obscure Brillouin signals and complicate measurements. To mitigate these limitations and ensure accurate optical analysis, thin tissue sections were prepared.

To ensure consistent measurement conditions, we selected a field of view within the reticular dermis at approximately 100 µm depth, where collagen fibres are thicker, more densely packed, and exhibit a more uniform alignment compared to the papillary dermis.

**SUPPLEMENTAL FIGURES:**

**
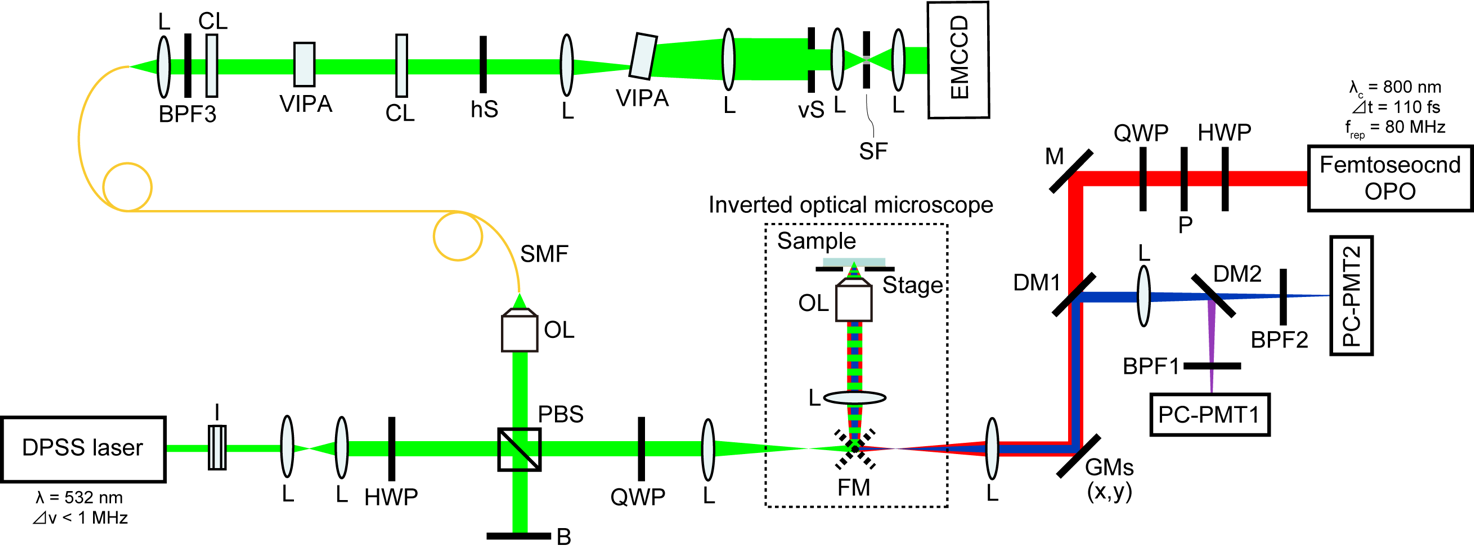
**

**Supplemental Figure 1. Optical setup for multimodal optical microscopy.** I: isolator; L: convex lens; HWP: half-wave plate; P: polarizer; PBS: polarization beam splitter; B: beam block; QWP: quarter-wave plate; FM: flipper mirror; OL: objective lens; SMF: single-mode optical fibre; CL: cylindrical convex lens; hS: horizontal slit; vS: vertical slit; SF: spatial filter; EMCCD: electron-multiplying CCD camera; M: mirror; GM: galvanometer mirror; DM: dichroic mirror; BPF: optical band-pass filter; PC-PMT: photon-counting photomultiplier.


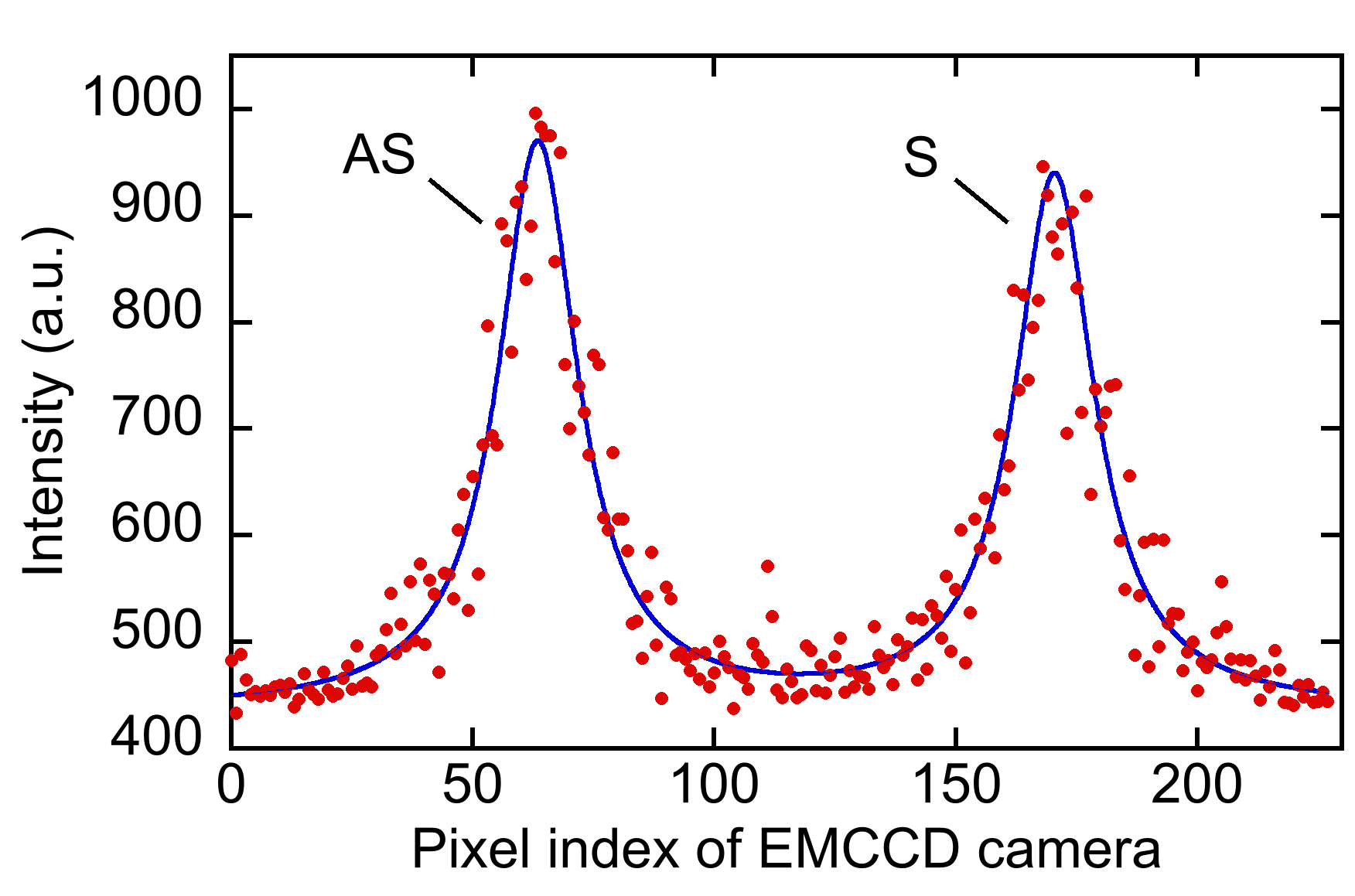


**Supplemental Figure 2. Typical Brillouin spectrum in human dermis.** Red dots represent the measured values, and the blue line indicate the nonlinear least-squares fit using the sum of two Lorentzian functions. AS: Anti-stokes, S: Stokes component of the Brillouin scattered light.

**
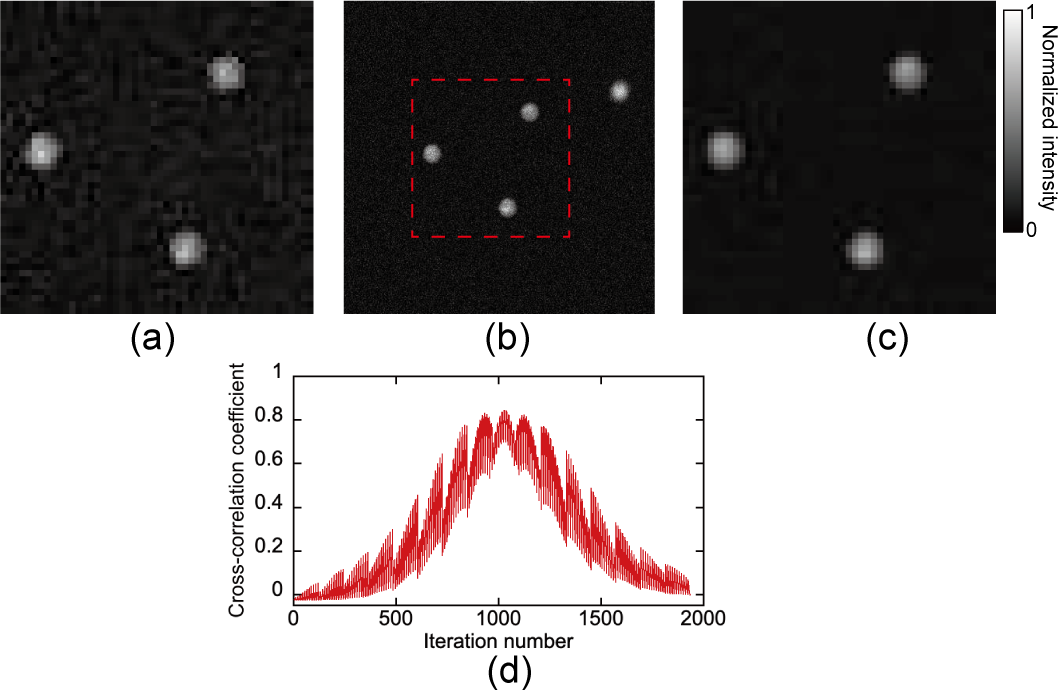
Supplemental Figure 3. 2D cross-correlation-based image registration.** (a) Fluorescence image acquired through the optical path of the Brillouin microscope (image size = 50 × 50 pixels). (b) TPEF image acquired by the multiphoton microscope (image size = 400 × 400 pixels). (c) Cropped and resized TPEF sub-image with the highest correlation, corresponding to the red dashed square in Fig. S3(b) (image size = 50 × 50 pixels). (d) Cross-correlation coefficient plotted over iterations, showing changes as the cropping starting coordinates (x, y) and scaling factor vary.

**References for SUPPORTING INFORMATION:**

[S1] Matsuzaki S, Hase E, Takanari H, Hayashi Y, Hayashi Y, Oshikata H, Minamikawa T, Kimura S, Ichimura-Shimizu M, Yasui T, Harada M, Tsuneyama K. Quantification of collagen fiber properties in alcoholic liver fibrosis using polarization-resolved second harmonic generation microscopy. Sci Rep. 2023;13:22100.

[S2] Scarcelli G, Yun SH. Confocal Brillouin microscopy for three-dimensional mechanical imaging. Nat Photonics. 2008;2:39.

[S3] Zhang J, Scarcelli G. Mapping mechanical properties of biological materials via an add-on Brillouin module to confocal microscopes. Nat Protoc. 2021;16:1251.

[S4] Zhang H, Asroui L, Randleman JB, Scarcelli G. Motion-tracking Brillouin microscopy for in-vivo corneal biomechanics mapping. Biomed Opt Express. 2022;13(12):6196-6210.

[S5] Romodina MN, Parmar A, Singh K. In vivo measurement of the biomechanical properties of human skin with motion-corrected Brillouin microscopy. Biomed Opt Express. 2024;15:1777.

[S6] Kabakova I, Zhang J, Xiang Y, Caponi S, Bilenca A, Guck J, Scarcelli G. Brillouin microscopy. Nat Rev Methods Primers. 2024;4:8. (Same reference as citation [6] in the main text.)

[S7] Schlüßler R, Kim K, Nötzel M, et al. Correlative all-optical quantification of mass density and mechanics of subcellular compartments with fluorescence specificity. eLife. 2022;11:e68490.

[S8] Bailey M, Alunni-Cardinali M, Correa N, Caponi S, Holsgrove T, Barr H, Stone N, Winlove CP, Fioretto D, Palombo F. Viscoelastic properties of biopolymer hydrogels. Sci Adv. 2020;6: eabc1937.

[S9] Brillouin Scattering Database. Available at: <http://koski.ucdavis.edu/BRILLOUIN/index.html>.

[S10] Mercatelli R, Mattana S, Capozzoli L, Ratto F, Rossi F, Pini R, Fioretto D, Pavone FS, Caponi S, Cicchi R. Morpho-mechanics of human collagen superstructures revealed by all-optical correlative micro-spectroscopies. Commun Biol. 2019;2:117. (Same reference as citation [7] in the main text.)
